# Millisecond Laser Oblique Hole Processing of Alumina Ceramics

**DOI:** 10.3390/nano15161261

**Published:** 2025-08-15

**Authors:** Yuyang Chen, Xianshi Jia, Zhou Li, Chuan Guo, Ranfei Guo, Kai Li, Cong Wang, Wenda Cui, Changqing Song, Kai Han, Ji’an Duan

**Affiliations:** 1 State Key Laboratory of Precision Manufacturing for Extreme Service Performance, College of Mechanical and Electrical Engineering, Central South University, Changsha 410083, China; 243711073@csu.edu.cn (Y.C.); likai01@csu.edu.cn (K.L.); wangcong@csu.edu.cn (C.W.); duanjian@csu.edu.cn (J.D.); 2 School of Intelligent Manufacturing, Hunan First Normal University, Changsha 410221, China; lit_ww@hnfnu.edu.cn; 3 College of Advanced Interdisciplinary Studies, National University of Defense Technology, Changsha 410083, China; guochuan20@nudt.edu.cn (C.G.); cuiwenda@nudt.edu.cn (W.C.); songchangqing08@nudt.edu.cn (C.S.); 4 Nanhu Laser Laboratory, National University of Defense Technology, Changsha 410083, China; 5 Hunan Provincial Key Laboratory of High Energy Laser Technology, National University of Defense Technology, Changsha 410083, China; 6 Bosch Automotive Products (Changsha) Co., Ltd., No.26, Lixiang Road Middle, Changsha 410100, China; ranfei.guo@cn.bosch.com

**Keywords:** alumina ceramic, millisecond laser, laser drilling, in situ imaging, theoretical simulation, defects control

## Abstract

Alumina ceramic substrates are ideal materials for next-generation microelectronic systems and devices, widely used in aerospace, 5G communications, and LED lighting. High-quality hole processing is essential for system interconnection and device packaging. Millisecond lasers have emerged as a promising choice for hole processing in alumina ceramic due to their high processing efficiency. However, existing research has rarely explored the mechanisms and processing techniques of millisecond laser oblique hole formation. This study systematically investigates the dynamic evolution of oblique hole processing in alumina ceramic through theoretical simulations, online detection, and process experiments. Through the simulation model, we have established the relationship between material temperature and hole depth. By analyzing the ablation phenomena on the upper and lower surfaces of the ceramic during the transient interaction process between the millisecond laser and the ceramic, the material removal mechanism in this process is elucidated. Additionally, this study examines the millisecond laser oblique hole processing technology by analyzing the influence of various laser parameters on hole formation. It reveals that appropriately increasing the single-pulse energy of millisecond lasers can optimize the material removal rate and hole taper. Ultimately, the formation mechanism of millisecond laser oblique hole processing in alumina ceramics is comprehensively summarized. The results provide theoretical and methodological guidance for high-speed laser drilling of alumina ceramic substrates.

## 1. Introduction

Alumina ceramic substrates are characterized by high hardness, thermal conductivity, electrical resistivity, and thermal stability, along with a low dielectric constant and a thermal expansion coefficient that matches that of chips [1,2,3]. These properties make them the preferred choice for next-generation microelectronic devices and systems. They have become the primary material for circuit substrates in fields such as aerospace, 5G communications, high-power semiconductor devices, and high-power LED lighting, offering broad application prospects [4,5,6]. In recent years, the rapid development of device miniaturization has driven the need for high-density interconnections in electronic systems. This necessitates the processing of alumina ceramic substrates with multiple sizes, spacings, and a high number of holes to provide internal circuit interconnections. Additionally, the processing quality must meet the packaging requirements for chip conduction and pin fixation.

Alumina ceramic is a typical hard and brittle material, and traditional mechanical processing methods are prone to causing substrate fractures. Special processing methods also face various limitations and have not been widely adopted [7,8]. With the technological maturity and widespread application of industrial lasers, laser processing technology has emerged as a new option for ceramic processing [9,10,11,12,13]. In existing research, picosecond and femtosecond lasers have demonstrated quality advantages in micron and nanoscale manufacturing [14,15,16,17,18,19]. However, they still struggle to meet the requirements for industrial-scale processing of ceramic substrates in terms of single-hole processing efficiency, stability, and equipment costs [13,19]. Nanosecond lasers, with their high peak power density, can easily lead to plasma shielding, limiting the energy coupled with the material. They often rely on spiral drilling to improve processing quality [20,21,22], but this process is time-consuming and offers limited processing efficiency (<5 holes/s). Millisecond lasers, with their large pulse energy, provide high material removal efficiency. However, the processing is accompanied by significant thermal effects, which can lead to quality defects such as large taper angles and noticeable cracks in the holes [23,24,25,26,27]. Therefore, current single-laser processing methods still face challenges in achieving both high efficiency and high-quality hole processing for alumina ceramic substrates [28,29,30].

In recent years, significant progress has been made in millisecond laser drilling of alumina ceramic. In 2012, M.M. Hanon et al. used an Nd:YAG laser to process alumina ceramic with thicknesses of 5 mm and 10.5 mm. The laser parameters used were 5–9 kW, 1–6 ms, and 5–20 Hz, and the diameter of the processed hole was 600–1200 μm. Through theoretical and experimental analysis, they studied the influence of various laser processing parameters on hole quality. Adjusting the focal plane of the laser was proven to effectively improve hole taper [31]. Yan et al. used a 3.5 kW, 100 μm, 0.35–3.5 kW, 5–100% duty cycle, 10–500 Hz gated CW laser to drill holes in alumina ceramic substrates with a thickness of 4.4 mm. The study investigates the effects of parameters such as laser peak power, pulse repetition rate, and pulse duty cycle on the drilling performance. Ultimately, holes with diameters ranging from approximately ~450 μm to ~1250 μm are machined using the CO_2_ laser, significantly larger than the diameter of the focused laser beam [24]. It is evident that the drilling processes involving Nd:YAG lasers and CO_2_ lasers are accompanied by significant thermal effects, leading to issues such as larger hole diameters and poorer taper quality. In contrast, the fiber lasers offer faster processing speeds and superior processing quality compared to other millisecond lasers. In 2015, B. Adelmann et al. investigated the use of a QCW fiber laser to process alumina ceramic substrates with a thickness of 0.635 mm. They optimized hole quality by adjusting laser process parameters. The results show that by using a laser power of 500 W, a drilling time of 200 ms, and an appropriate focal position, holes with near-zero taper, good roundness, and diameters in the tens of micrometers could be achieved [5]. In 2017, L. Rihakova and H. Chmelickova compared the performance of a QCW fiber laser with an Nd:YAG laser in processing alumina ceramic. The lasers used had a wavelength of 1064 nm, a repetition rate of 50 Hz, and an energy of 0.34–1.54 J. Ultimately, the holes produced by the QCW fiber laser were smaller in diameter (133–266 μm) than those produced by the Nd:YAG laser (350–600 μm) [32]. Although the aforementioned studies have successfully produced high-quality holes using different lasers, they have not yet provided a detailed elucidation of the formation mechanisms underlying quality defects in millisecond laser drilling of alumina ceramic. Therefore, the hybrid laser processing method can be actively employed to regulate drilling quality, achieving high-efficiency and high-precision hole processing.

To achieve high-efficiency and high-quality hole processing on alumina ceramic substrates, research has focused on reducing the thermal effects associated with millisecond laser processing. Various hybrid laser processing techniques [33,34,35,36,37], such as electric furnace heating [38], flame ablation heating [39], plasma ablation heating [40], and combined pulse laser drilling [41,42,43,44,45,46], have been explored. These methods aim to reduce thermal stress during millisecond (or continuous wave (CW)) laser processing by preheating or post-heating, thereby controlling crack formation in alumina ceramic. Additionally, static water-assisted methods [47,48,49] have been proven to mitigate the thermal effects of millisecond laser drilling, improving hole cracks and taper [48,50,51,52,53,54,55]. However, these processing methods are relatively complex and still struggle to meet the single-hole processing efficiency requirements. In recent years, significant progress has been made in high-pressure gas-assisted millisecond hybrid laser processing technology. Using a single-pulse drilling method, processing speeds of up to 300 holes/s (on 0.635 mm thick substrates) have been achieved. Nevertheless, due to the thermal effects of millisecond laser processing, the diameter difference between the upper and lower surfaces of the holes exceeds 50%, and the taper cannot be effectively controlled [56,57,58]. Therefore, millisecond lasers, due to their high material removal efficiency, are an effective choice for high-speed hole processing of alumina ceramic substrates [46,59,60]. However, the significant thermal effects associated with their processing, which often lead to quality defects such as cracks and taper, represent a critical challenge that needs to be addressed. Therefore, it is essential to conduct systematic research on the key technologies and scientific issues involved in millisecond laser drilling of alumina ceramic, laying a theoretical foundation for process innovation and equipment development.

In this context, model simulation and shadow imaging technology are important methods for studying the mechanism of laser hole formation in alumina ceramic [61,62,63,64,65]. Through model simulation, the effects of various laser parameters (such as peak power, ablation time, duty cycle, repetition rate, etc.) on processing efficiency can be explored, and the temperature distribution and changes under different laser parameter irradiations can be analyzed [24]. In 2023, Li et al. used a two-phase flow and level set method to construct a model for laser drilling of alumina ceramic, comparing the differences in hole quality and efficiency between millisecond lasers and CPL (combined pulse laser) [66]. In 2019, Qin et al. established a numerical model based on the Navier–Stokes equations to simulate the drilling process using millisecond and nanosecond composite pulse lasers, studying the hole depth, molten pool expansion, and hole geometry under different laser parameters [67]. The aforementioned theoretical studies have elucidated the mechanism of millisecond laser drilling of straight holes in detail through establishing numerical models. However, there is a paucity of theoretical research addressing the mechanism of oblique hole drilling [68].

On the other hand, high-speed shadow imaging technology, based on high-speed cameras, utilizes changes in the refractive index of the medium to image and observe the laser damage process in materials [69]. It allows for continuous multiple observations of the same phenomenon, capturing the dynamic process, and is an important tool for investigating the transient processes of laser–material interactions [62,67,70,71]. In 2023, Jia et al. systematically studied the mechanism of high-energy laser ablation of alumina ceramic, using high-speed shadow imaging to investigate the transient processes between high-energy lasers and ceramic targets [58]. The aforementioned studies have primarily focused on the ablation phenomena occurring on the upper surface of alumina ceramic during the millisecond laser hole formation process, overlooking the phenomena on the lower surface of the alumina ceramic. During the interaction between the laser and the material, it is inevitable that a recast layer blockage may occur, rendering the material removal process unobservable on the ceramic’s upper surface. Due to the transparency of molten alumina ceramic, the laser can continue processing through the blocked recast layer. Therefore, once the hole in the ceramic is blocked, the molten material is removed from the bottom. This indicates that the study of the material removal process requires a combined analysis of the ablation phenomena on both the upper and lower surfaces.

Our previous research primarily focused on the straight hole processing of alumina ceramic [43,58,66], with limited exploration of oblique hole processing. The oblique hole processing of alumina ceramic finds extensive applications across various fields. In the microelectronics sector, oblique holes in ceramic substrates can serve for thermal dissipation or electrical signal transmission purposes [72]. In the medical field, oblique holes in ceramic endoscopes enable drug delivery applications [73]. For aerospace applications, oblique holes in ceramic injectors can optimize fuel mixing efficiency [74]. Compared to straight hole drilling, millisecond laser oblique hole processing of alumina ceramic not only faces common challenges such as excessive thermal effects leading to issues like increased crack formation and poor taper quality, but also must address additional difficulties inherent to angled processing. These include increased effective drilling depth due to the inclined angle and the heightened challenge of molten material removal within the holes.

In this regard, this paper establishes a numerical model for the oblique hole processing of alumina ceramic using millisecond lasers, investigating the physical changes during the interaction between millisecond lasers and ceramic materials. Compared to prior research in millisecond laser micro-hole formation, this work innovatively proposes employing high temporal resolution shadowgraph imaging to observe ablation phenomena on both upper and lower surfaces of alumina ceramic during oblique hole processing. By integrating simulation and imaging results, we correlated the simulated temperature curve with the ablation phenomenon captured by high-speed cameras, comprehensively elucidating the mechanism of oblique hole machining in alumina ceramics. Finally, process experiments based on theoretical findings were conducted. We characterized the upper- and lower-hole surfaces via optical microscopy to systematically examine the influence of key laser parameters (such as peak power, ablation time, duty cycle, and repetition rate) on machining quality and efficiency. In summary, this paper systematically investigates the oblique hole processing with millisecond lasers from the perspectives of simulation, imaging, and process experiments, comprehensively revealing the hole formation mechanism in alumina ceramic and providing a reference for high-quality oblique hole processing.

## 2. Materials and Methods

This section details the research methodology. First, a preliminary investigation into the mechanism of millisecond laser oblique hole processing in alumina ceramics is conducted using a theoretical model. Subsequently, alumina ceramic samples are processed using a millisecond laser under varying parameters, and we used a high-speed camera to simultaneously record the material removal process on both the upper and lower surfaces of the sample for the first time. Finally, the surface morphology of the processed samples is microscopically characterized. By integrating fluid dynamics simulations, high temporal resolution shadow imaging, and microscopic characterization, not only can different parameter laser drilling morphology rules be predicted from theoretical simulation, but also the drilling mechanism and feasibility of the verification model can be explored from the actual experimental results. The two mutually verify and explain each other, and the mechanism of millisecond laser oblique hole processing in alumina ceramics is systematically elucidated. This research approach can provide new strategies for multi-species hole drilling and its defects control.

### 2.1. Finite Element Simulation

This study establishes a mathematical model of millisecond laser ablation based on heat transfer and fluid dynamics, further revealing the ablation mechanism of millisecond lasers on alumina ceramic. The physical changes during the interaction between the laser and the material are described using the Navier–Stokes equations [75]:

(1) $$\nabla \cdot \vec{u}=0,$$

(2) $$\rho \frac{\partial \vec{u}}{\partial t}+\rho (\vec{u}\cdot \nabla )\vec{u}=-\nabla \cdot [pI+\mu (\nabla \vec{u}+{\left(\nabla \vec{u}\right)}^{T})]+\rho g+\vec{F_{bf}},$$

(3) $$\rho C_{P}\frac{\partial T}{\partial t}+\rho C_{P}\vec{u}\cdot \nabla T+\nabla \cdot (-k\nabla T)=Q_{laser\_ms}-Q_{loss},$$

where in the equations, $\vec{u}$, μ, and ρ represent fluid velocity, dynamic viscosity, and density, respectively, *C_p_* and *k* denote specific heat capacity and thermal conductivity, and ρg and $\vec{F_{bf}}$ correspond to gravity and volume force. *Q_laser_ms_* denotes the energy absorption during laser–material interaction, while *Q_loss_* represents the energy loss in the same process. The spatial energy distribution of the laser heat source *Q_laser_ms_* follows a Gaussian distribution, expressed as:

(4) $$Q_{laser\_ms}=\left\{\begin{array}{cc} 0 & t\notin t_{ms}\\ \alpha_{ms}\frac{P_{ms}}{\pi R_{ms}}\cdot \exp\left(-\frac{y^{2}}{R_{ms}^{2}}\right)\delta (\phi ) & t\in t_{ms} \end{array}\right.,$$

In the equation, $\alpha_{ms}$, $P_{ms},$ and $R_{ms}$ represent the absorptivity of alumina ceramics to millisecond laser irradiation, the peak power of the millisecond laser, and the laser beam radius, respectively. Among them, the value of $\alpha_{ms}$ is determined by measuring the absorptivity of a black-coated alumina ceramic sample at 1080 nm using a spectrophotometer [60]. The $\delta \left(\phi \right)$ function is introduced to ensure that the laser energy acts on the gas–liquid interface, while $t_{ms}$ denotes the operational range of the millisecond laser. The $\delta \left(\phi \right)$ function is expressed as [76]:

(5) δ(ϕ)=6|ϕ(1−ϕ)||∇ϕ|,

Furthermore, the energy loss denoted by parameter *Q_loss_* in the equation comprises heat dissipation through thermal radiation, thermal convection, and the latent heat of evaporation. This relationship is expressed as:

(6) $$Q_{loss}=-(L_{v}\dot{m}+\sigma_{0}k_{B}(T^{4}-T_{v}^{4})+h(T-T_{v}))\delta (\phi ),$$

In the equation, the first term corresponds to the energy loss due to the latent heat of vaporization during the evaporation of molten pool material, and *L_v_* and $\dot{m}$ represent the latent heat of vaporization and the lost mass. The second term represents thermal radiation loss from the molten pool, and $\sigma_{0},\;k_{B},$ and $T_{v}$ represent the radiation emissivity, the Boltzmann constant, and the vaporization point, respectively. The third term accounts for thermal convection loss from the molten pool, and h represents the convective heat transfer coefficient. The introduction of the $\delta \left(\phi \right)$ function ensures that energy loss occurs specifically at the gas–liquid interface.

The Level-Set method is employed to track the gas–liquid interface. To ensure the model operates effectively, initialization and interface transport are incorporated into the original equations, resulting in a general equation that can be expressed as [76]:

(7) $$\frac{\partial \phi }{\partial t}+\vec{u}\cdot \nabla \phi +\gamma \left[\left(\nabla \cdot \left(\phi (1-\phi )\frac{\nabla \phi }{\left|\nabla \phi \right|}\right)\right)-\varepsilon \nabla \cdot \nabla \phi \right]=0,$$

in the equation, γ and ε are Level-Set parameters: γ determines the reinitialization amount of the equation, ensuring that the Level-Set function maintains its signed distance property over time. ε corresponds to half the thickness of the interface, which is typically determined by the maximum size of the grid used for discretization, influencing the numerical stability and accuracy of the interface tracking. ϕ(x,y,t) represents the Level-Set function. When x < −ε, ϕ = 0, it indicates that the material in the defined domain is in the solid state; when x > ε, ϕ = 1, it indicates the material is in the gaseous state; and when −ε ≤ x ≤ ε, ϕ = 0.5, it represents the gas–liquid interface within the molten pool [77].

Using the Heat Transfer in Fluids and Laminar Two-Phase Flow, Level Set modules in COMSOL 6.1 software, a two-dimensional numerical model for millisecond laser oblique hole processing of alumina ceramic is established. This model simulates the phase transition of alumina ceramics from solid to liquid and finally to gaseous state during computation. Thermophysical parameters such as specific heat capacity and thermal conductivity vary with function ϕ(x,y,t). As temperature increases, function ϕ(x,y,t) transitions smoothly from 0 to 1, enabling gradual property interpolation between solid and liquid phases.

A free triangular mesh is employed for model construction. In order to simplify the model calculation, the laser irradiation zone is selectively refined, non-irradiated zone utilizes coarser meshing, featuring a maximum element size of 9.2 × 10^−2^ mm and minimum size of 2.0 × 10^−3^ mm. The resultant mesh contains approximately 5000 elements.

The boundary conditions of the model must account for discrepancies between the simulation environment and actual machining conditions. To better align with real-world processing, the initial temperature of both the alumina ceramic and ambient air is set to 293.15 K. Thermal exchange between the model and surrounding environment is incorporated during simulation by applying convective heat flux boundary conditions to all external surfaces.

**Figure 1:**
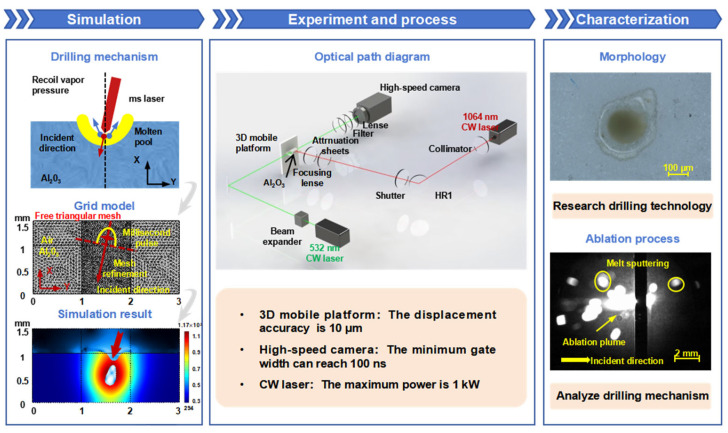
The schematic diagram outlines the research methodology for millisecond laser oblique hole drilling in alumina ceramics. Simulation: Depicts the model development process and simulation results for millisecond laser oblique hole drilling in alumina ceramics. Experiment and process: Illustrates the schematic of a shadow imaging system used for monitoring the oblique hole drilling process with millisecond lasers. Characterization: Shows surface microtopography images of the processed alumina ceramics and shadowgraphs of ablation phenomena captured during the machining process.

A Gaussian-distributed millisecond laser is applied at the interface between alumina ceramic and air to study material flow and temperature distribution during the laser ablation process. The laser is incident at an angle of 11 degrees to the surface normal of the ceramic, with a power of 600 W and a radius of 55 μm. The simulation results, as shown in Figure 1, demonstrate that under the sputtering effect of recoil pressure, the molten material forms a closed hole at the center. The material parameters of alumina ceramic used in the numerical analysis are listed in Table 1.

In this study, the millisecond laser repetition rate is simulated in the range of 50–500 Hz. By varying the repetition rate, the temperature changes, temperature distribution, and hole evolution processes under different repetition rates are investigated. This provides strong evidence for understanding the mechanism of millisecond laser oblique hole processing in alumina ceramic.

Figure 1 presents the mesh diagram of a two-dimensional numerical simulation model for millisecond laser oblique hole processing of alumina ceramic. The mesh is refined in the laser processing area to accurately capture the thermal and fluid dynamics phenomena during ablation. The energy distribution of the millisecond laser on the alumina ceramic is also illustrated, highlighting the localized heating and material removal processes. A schematic diagram of the millisecond laser oblique hole processing corresponding to the mesh diagram is provided in Figure 1.

### 2.2. Laser Drilling System

The experimental system described in this paper can be divided into two optical paths: one for processing and the other for monitoring, as illustrated in Figure 1. The processing optical path provides millisecond laser processing of the sample. A single-mode CW fiber laser (1080 nm, NA:0.06, DG laser, Mianyang, China) is employed, with operational limits of 1000 W maximum power and 5 kHz maximum modulation rate. The core diameter of the CW laser fiber is 12 μm. The CW laser is irradiated onto an alumina ceramic sample placed on a three-dimensional displacement platform after passing through several mirrors. The processing optical path is at an 11-degree angle to the normal of the sample’s upper surface. The monitoring optical path is responsible for providing background light, using a CW laser with a wavelength of 532 nm. Firstly, the laser beam is expanded by a beam expander (6×), then adjusted to the height of the sample by several mirrors and made parallel to the sample to traverse the sample surface. Subsequently, the light path passes through an attenuator, and finally, the collected information is magnified by a lens with a focal length of 60 mm and imaged into a high-speed camera (FASTCAM NOVA S9, Japan). The camera’s frame rate is set at 10,000 fps, with a shutter speed of 1/10,000 s, and the delay between the high-speed camera and the laser is controlled by the DG645. The zero-time image captured is shown in Figure 1. The laser parameters used in the experiment are shown in Table 2.

### 2.3. Materials Preparation

The experimental sample used in this study was alumina ceramic (96% Al_2_O_3_). It has a thickness of 3 mm with a size of 30 mm × 30 mm. Its surface exhibits high reflectivity and a certain degree of transmissivity, making it difficult to achieve effective ablation with millisecond lasers. Therefore, surface pretreatment is necessary to enhance the laser absorptivity of the surface. A black pigment solution was prepared using Marley’s black Gouache, into which the alumina ceramic was immersed [58]. The mixture was stirred with a glass rod to ensure that an even coating of black particles adhered to the ceramic’s surface. Subsequently, the ceramic was removed from the solution and placed horizontally, with absorbent paper used to soak up any excess liquid from the surface. Once dried, the sample was coated with a uniform layer of black, which increases the laser absorptivity without affecting the surface roughness of the sample.

## 3. Results

This section employs fluid dynamics simulations, high temporal resolution shadow imaging, and microscopic characterization for comprehensive analysis of the experimental results. By using a theoretical–imaging–experimental combination method, the interaction process between alumina ceramics and a millisecond laser was systematically explored, and the evolution of hole morphology, temperature changes in the processing zone, and the material removal process of molten material during millisecond laser oblique hole processing of alumina ceramics were explained in detail. The three complement and verify each other, fully explaining the forming mechanism of millisecond laser oblique hole processing in alumina ceramics. Furthermore, building on the conclusions drawn from the aforementioned analyses, process experiments are conducted to examine the variations in hole diameter and taper of the oblique hole under different laser parameters.

### 3.1. Theoretical–Imaging–Experimental Comprehensive Analysis

This subsection employs an integrated theoretical–imaging–experimental methodology to analyze the oblique hole processing using a 200 Hz repetition rate millisecond laser. The fluid dynamics simulation is utilized to investigate the variations in temperature, molten pool morphology, and hole depth during the oblique drilling process. High-temporal-resolution shadowgraph imaging complements the analysis by capturing dynamic material removal mechanisms, while experimentally obtained oblique hole morphologies validate the simulation results. These three approaches synergistically elucidate the interaction mechanism between alumina ceramic and a millisecond laser. Finally, the fluid dynamics model is applied to simulate oblique hole processing under different repetition rates, providing critical references for subsequent experimental parameter optimization.

**Figure 2:**
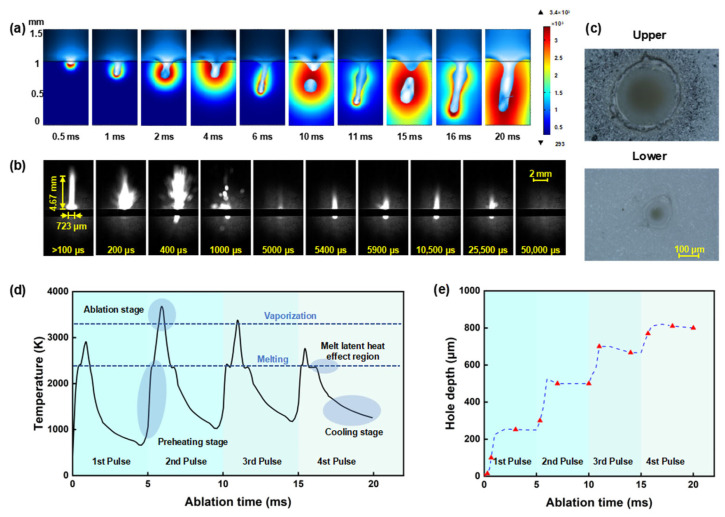
Numerical simulation, shadow imaging, and ablation zone morphology of millisecond laser oblique hole processing in alumina ceramic: (**a**) Temperature contour maps of the hole formation process; (**b**) Shadow sequence images of alumina ceramic processed by millisecond lasers at a repetition rate of 200 Hz; (**c**) Upper- and lower-surface morphology at a repetition rate of 200 Hz; (**d**) Temperature changes during the hole formation process. (**e**) Simulated depth changes at different ablation times.

To study the mechanism of millisecond laser hole formation in alumina ceramic, simulations are conducted using a millisecond laser with a power of 600 W, a repetition rate of 200 Hz, and a duty cycle of 20%. Figure 2 presents the simulated results of alumina ceramic hole formation at different ablation times, with each 5 ms cycle consisting of 1 ms of laser processing followed by 4 ms of laser shutdown for material cooling. From Figure 2a, it can be observed that during the laser irradiation time, the hole depth increases with processing time. During the cooling phase, as the molten material within the ablation zone flows, the hole depth decreases slightly, and the hole morphology undergoes significant changes during this period.

Figure 2d presents the temperature curve of our simulated process. By studying the temperature variations, we can better reveal the mechanism of millisecond laser hole formation in alumina ceramic. The temperature curve exhibits periodic changes, with each cycle’s ablation process divided into preheating, ablation, and cooling stages. In the first cycle, the temperature rapidly rises upon laser irradiation and reaches the melting point of alumina ceramic. However, due to the latent heat of melting, the temperature remains near the melting point for an extended period [78]. During this stage, the alumina ceramic surface gradually melts, forming a molten pool. Subsequently, the temperature continues to rise to near the evaporation point, and a hole structure begins to form at the bottom of the molten pool. In the following two pulses, since the ceramic has already developed a hole structure, it absorbs laser energy more efficiently. The temperature in the ablation zone quickly transitions through the heating phase and continues to rise above 3500 K. During this rise, the temperature occasionally drops, due to the removal of high-temperature molten material, which carries away some heat, causing a temporary decrease in the molten pool temperature [58]. In the fourth cycle, as the hole has reached a significant depth, laser energy experiences greater losses during transmission, leading to lower achievable temperatures in the ablation zone [12]. After each laser shutdown, the process enters the cooling phase. During cooling, due to the excessive hole depth, some molten material cannot be fully removed, and the residual molten material tends to cause blockages within the hole under the influence of molten pool flow. Additionally, due to the latent heat of melting, the temperature curve remains near the melting point for a period before continuing to decrease [79].

Figure 2e shows the variation in hole depth at different ablation times. Combined with the temperature change curve (Figure 2d), it is evident that the first and fourth cycles achieve lower temperatures, resulting in slower changes in hole depth, while the second and third cycles reach higher temperatures, representing the intense ablation phase, during which the hole depth increases rapidly. During the cooling phase of each cycle, the hole depth slightly decreases as some molten material flows back. Additionally, residual heat during the cooling process continues to expand the molten pool diameter, which is particularly noticeable when the hole becomes blocked.

To further analyze the dynamic interaction between the sample and the laser, high-speed shadow imaging technology is employed to meticulously examine the ablation process at a repetition rate of 200 Hz. Through the analysis of the captured images (Figure 2b), the millisecond laser ablation process can be delineated into three distinct phases: the preheating phase, the intense sputtering phase, and the final stable ablation phase. The millisecond laser, operating at 200 Hz, underwent a total of 10 pulses within a 50 ms operational timeframe. During the initial pulse ablation, the sample first experiences the preheating phase, wherein a stable laser-supported combustion wave (LSCW) forms on the sample’s surface without any sputtering of molten material [79]. This LSCW measured 4.67 mm in height and 723 μm in width. Correlating with the temperature profile from the simulation, the sample’s temperature is inferred to rise from ambient to 2350 K during this phase. Following the formation of the LSCW, the millisecond laser energy is effectively absorbed by the sample, causing the temperature to continue rising past the latent heat of melting, resulting in the appearance of a molten pool morphology on the sample’s surface. After sustained heating for 200 μs, the molten pool reaches the boiling point, at which the sputtering of molten material from the target surface becomes observable. Upon the effective removal of some molten material, a blind hole structure forms on the sample’s surface. After multiple reflections from the hole walls, the absorptivity of the sample to the millisecond laser further increases. At this juncture, the millisecond laser ablation enters the intense sputtering phase, occurring between 300 μs and 500 μs, where the material removal efficiency is at its peak. The temperature of the molten pool is presumed to be above the boiling point, with the hole depth rapidly increasing, signaling the onset of the intense ablation phase. At a delay of 400 μs, sputtering phenomena can be observed at the bottom of the alumina ceramic, indicating that the ceramic sample has been penetrated. In subsequent pulses, the sputtering phenomena significantly diminish, and the ablation transitions into the later stage of stable ablation. As the sample has developed a through hole, the majority of the laser energy escapes from the bottom of the oblique hole, and the temperature is below the melting point. The shadow images reveal scarcely any noticeable sputtering, with only a faint LSCW being observed.

To simplify computations, this model neglects shock wave effects during millisecond laser oblique hole processing. Consequently, significant deviations exist between simulated versus actual material removal efficiency and through-hole formation rates. By integrating model simulations with shadowgraph imaging results, general mechanisms governing millisecond laser micro-hole machining can be elucidated. When the molten pool temperature remains below the boiling point, alumina ceramics undergo a preheating phase without material ejection. During this stage, the LSCW forms on the surface, enhancing laser energy absorption. Upon exceeding the boiling point, the system transitions into an intensive ejection phase characterized by substantial expulsion of molten material from the pool, accompanied by rapid increases in oblique hole depth. Following through-hole formation, alumina enters a stable ablation phase where liquid spatter and LSCW diminish, and temperature gradually decreases. Critically, after initial pulse processing, the preexisting molten pool structure exhibits elevated absorptivity. Consequently, during subsequent pulses, the preheating phase shortens significantly, allowing temperature to rise more rapidly above the boiling threshold, as illustrated in Figure 2b,d.

Figure 2c depicts the morphology of the hole on the upper and lower surfaces after 50 ms of millisecond laser ablation at a repetition rate of 200 Hz. The diameter of the upper surface of the oblique hole is 155 μm, and the diameter of the lower surface is 64 μm, with a taper of 1:12. It is evident that the oblique holes produced using millisecond laser processing at these parameters exhibit a favorable taper. Analysis combined with simulation results indicates that at a repetition rate of 200 Hz, the energy per laser pulse is relatively low, the duration of the intense ablation phase is brief, and there is a substantial cooling period following each pulse, which significantly aids in reducing the thermal effects associated with processing. It is particularly noteworthy that the laser heat source in this model primarily acts on the bottom of the hole. Different from the actual situation, the mechanism of multiple reflections from the hole walls is neglected, which may result in an excessively small bottom diameter and inconsistent hole taper. However, during the cooling phase, with the assistance of molten pool flow, the bottom diameter gradually increases, and the hole taper improves, resulting in a final hole morphology that is similar to the actual situation.

**Figure 3:**
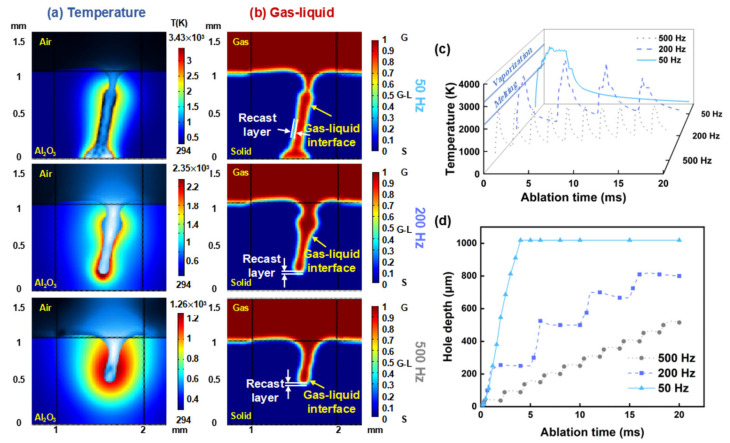
Numerical simulation results of oblique hole processing in alumina ceramic using millisecond lasers at different repetition rate: (**a**) Temperature contour maps of millisecond laser hole formation; (**b**) Gas–liquid volume fraction maps of millisecond laser hole formation; (**c**) Temperature changes during hole formation at different repetition rate; (**d**) Depth changes during hole formation at different repetition rate.

Furthermore, the computational results of the model under different repetition rates are compared. The laser parameters used are a power of 600 W, an ablation time of 20 ms, a duty cycle of 20%, and repetition rates of 50 Hz, 200 Hz, and 500 Hz. Figure 3a shows the temperature contour maps for each repetition rate. From Figure 3a, it can be observed that the oblique hole morphology produced by millisecond laser processing varies significantly with repetition rate. The depth of the oblique hole is negatively correlated with the repetition rate. At a repetition rate of 50 Hz, the millisecond laser has already produced a through hole within the 20 ms processing time, whereas at 500 Hz, the oblique hole depth only reaches half the thickness of the alumina ceramic. This aligns with the earlier conclusion that increasing single-pulse energy enhances the material removal rate (MRR). Notably, at a repetition rate of 50 Hz, a “hole closure” phenomenon occurs at the upper part of the oblique hole [25]. This is likely due to the excessive single-pulse energy causing an accumulation of molten material within the hole. As the hole depth increases, the saturated vapor pressure inside the hole is insufficient to expel all the molten material. The remaining molten material flows to the hole entrance under recoil pressure and solidifies upon cooling, forming a recast layer and resulting in the “hole closure” phenomenon.

Figure 3b shows the gas volume fraction maps for each repetition rate. In the figure, the red regions represent the gaseous state, while the blue regions represent the solid state. The volume fraction, ranging from 0 to 1, indicates the gradual transition of alumina ceramic from solid to gas. In the model, the gas–liquid interface of the molten pool is defined by the boundary where the gas volume fraction is 0.5, located between yellow and cyan in the figure [66]. The recast layer thickness is defined within the interface range of 0.4 to 0.6. From Figure 3, the recast layer thickness for each repetition rate can be determined. As the repetition rate increases, the single-pulse energy of the millisecond laser decreases, and the accompanying thermal effects are reduced. Consequently, the recast layer becomes thinner with the increasing repetition rate.

Figure 3c shows the temperature curves obtained at various repetition rates, where the periodic changes in the temperature curves correspond to the repetition rate. At a repetition rate of 50 Hz, the laser ablation process undergoes only one preheating phase, whereas at a repetition rate of 500 Hz, the process includes ten preheating phases as the laser turns on and off. Reflected in the temperature curves, at 50 Hz, after experiencing the preheating phase, the temperature continues to rise and eventually fluctuates around 3500 K. In contrast, at 500 Hz, the temperature curve repeatedly undergoes the preheating phase with each laser pulse cycle, with a significant amount of energy consumed by the latent heat of melting, resulting in a maximum temperature of only 2500 K. Figure 3d shows the curves of hole depth over time at various repetition rates. Combined with the temperature curves, it is evident that the change in hole depth is closely related to the temperature of the molten pool. During the laser irradiation time, the higher the temperature of the molten pool, the faster the rate of change in hole depth. The phase during which the molten pool temperature exceeds the boiling point is defined as the intense ablation phase. At a repetition rate of 500 Hz, the ablation process does not experience an intense ablation phase; at 200 Hz, the intense ablation phase lasts approximately 0.5 ms; and at 200 Hz, the intense ablation phase extends up to 2 ms.

In summary, although the total energy of the laser is the same, due to differences in energy per pulse and repetition rate, the intensity of ablation and the duration of the intense ablation phase during the oblique hole processing vary significantly. Therefore, millisecond lasers at a lower repetition rate possess superior ablation capabilities.

### 3.2. Influence of Millisecond Laser Repetition Rate on Oblique Hole Processing Performance

**Figure 4:**
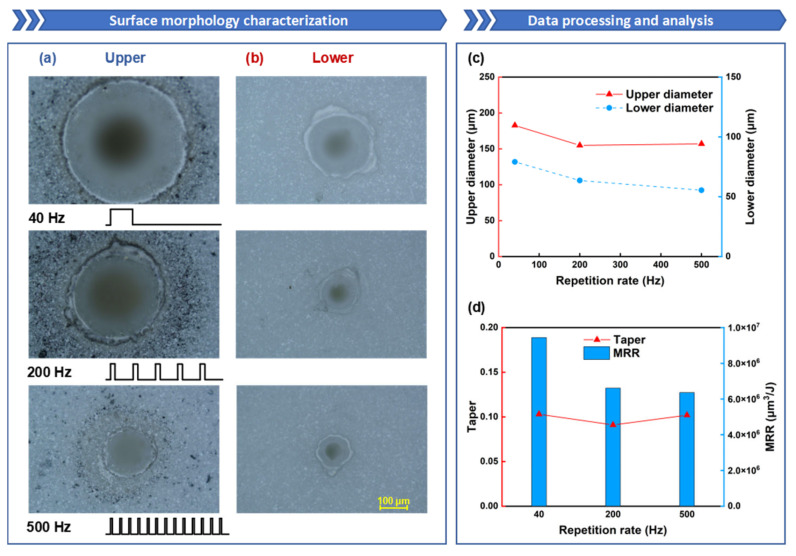
The influence of repetition rate on oblique hole processing performance: the repetition rate is set to 40 Hz, 200 Hz, and 500 Hz, while the other parameters are fixed: power of 600 W ablation time of 50 ms and duty cycle of 20%: (**a**) Upper-surface morphology; (**b**) Lower-surface morphology; (**c**) Upper- and lower-surface diameter line chart; (**d**) Taper and ablation efficiency comparison.

Firstly, building upon the foundation of model simulation, the influence of repetition rate on the quality and formation process of oblique hole processing is investigated through process experiments and shadow imaging technology. As can be seen from Figure 4a,b, there is an ablation zone around the hole, which is a molten pool structure formed on the surface of the alumina ceramic under the action of the laser. The formation of the ablation zone is related to the coupling process between the millisecond laser and the surface of the alumina ceramic. With the aid of the surface black coating, the alumina ceramic effectively absorbs the millisecond laser through the transfer of deposited coating particles. Assisted by the absorption coating, the millisecond laser can gradually create defects on the surface of the alumina ceramic through ablation, thereby enhancing the absorptivity of the ceramic to the millisecond laser. As the laser energy continues to be absorbed, the temperature rises to the melting point, and the alumina ceramic begins to melt, forming a molten pool. The absorptivity of the material in the molten state to the laser increases, and the molten pool deepens further. Meanwhile, the rise in temperature causes the material to vaporize, generating vapor pressure within the molten pool. Under the action of the recoil pressure in the thermal stress field, the material is expelled in the form of gas or ejected as liquid splashes, forming the hole structure.

Figure 4a,b display the morphology of the upper and lower surfaces at different repetition rates. Under the condition of the same total energy, a higher repetition rate means lower single-pulse energy and higher pulse rate, leading to a more uniform distribution of laser energy over time; a lower repetition rate means higher single-pulse energy and lower pulse rate, resulting in a more concentrated distribution of laser energy over time. Higher single-pulse energy provides better material removal efficiency, so as the repetition rate increases, the diameters of both the upper and lower surfaces tend to decrease; the diameter of the upper surface decreased from 182 μm to 157 μm, and the diameter of the lower surface decreased from 79 μm to 55 μm, as shown in Figure 4c.

As shown in Figure 4d, the taper initially decreases and then increases with the rise in repetition rate. When the repetition rate increases from 40 Hz to 200 Hz, the hole taper shows a downward trend, likely because, as the repetition rate increases, the laser energy is distributed more evenly over time, leading to a more gradual change in diameter and an improvement in the hole taper. However, when the repetition rate increases from 200 Hz to 500 Hz, the hole taper shows an upward trend, possibly due to the single-pulse energy being too low to effectively remove material at the bottom, causing a rapid decrease in the lower-surface diameter and resulting in a poorer hole taper. The MRR decreases as the repetition rate increases, with the material removal rate reaching its highest at 9.44 × 10^6^ μm^3^/J at a repetition rate of 40 Hz. As the repetition rate increases, the MRR gradually decreases. It can be summarized that under the same total energy, higher single-pulse energy can lead to higher MRR.

The calculation formula for taper C is as follows [80]:

(8) $$C=\frac{d_{upper}-d_{lower}}{L},$$

In the formula, $d_{upper}$ represents the diameter of the upper surface, $d_{lower}$ represents the diameter of the lower surface, and L represents the length of the oblique hole.

**Figure 5:**
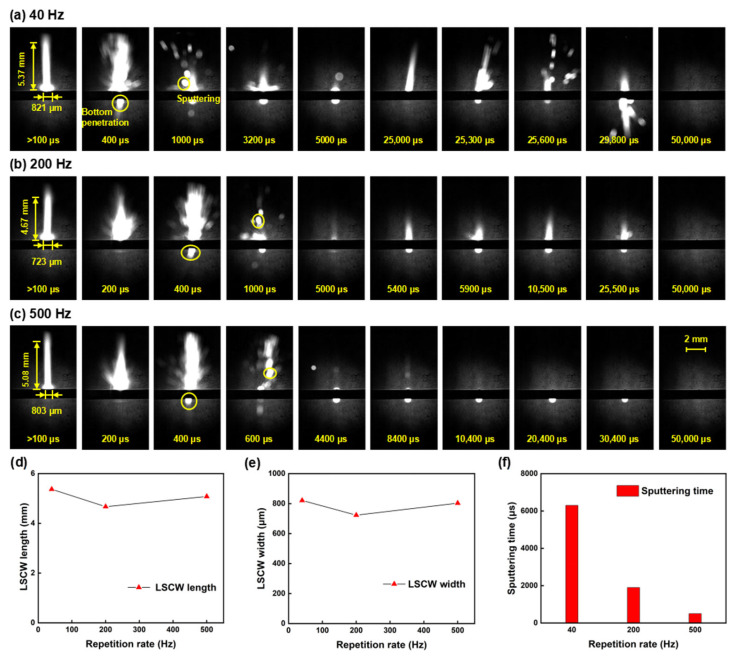
Shadow sequence images of alumina ceramic processed by millisecond lasers at different repetition rates: (**a**) 40 Hz; (**b**) 200 Hz; (**c**) 500 Hz; (**d**) LSCW length line chart; (**e**) LSCW width line chart; (**f**) Sputtering time bar chart.

Figure 5a–c present shadow sequence images at various repetition rates. As illustrated in Figure 5d,e, the morphology of the LSCW shows little variation across different repetition rates, which may be attributed to the consistent laser power under each operating condition, resulting in a similar amount of energy absorbed by the alumina ceramic per unit time. However, an increase in repetition rate implies a decrease in single-pulse energy, indicating that the ablation area will gradually diminish. By correlating the changes in diameter with repetition rate, it is observed that although the average laser power and ablation time are consistent across different repetition rates, the time to reach the ablation threshold varies significantly. This results in a notably larger ablation range and higher MRR at lower repetition rates compared to higher ones. Furthermore, a comparison of the sputtering phenomena at different repetition rates is conducted. Since the laser energy absorbed by the sample per unit time is consistent, the initiation time of sputtering and the time at which sputtering occurs at the bottom during the first pulse are essentially the same across different repetition rates. As the repetition rate increases and single-pulse energy decreases, both the duration and intensity of sputtering also decline, as shown in Figure 5f, aligning with the earlier observation that MRR decreases with increasing repetition rates. Under high repetition rate conditions, sputtering phenomena are difficult to observe in subsequent pulses, not only due to lower single-pulse energy but also because the interval between consecutive pulses is too short for the melt to cool and form a recast layer. Since molten alumina is transparent, this means that most of the subsequent laser energy escapes from the hole, manifesting in the shadow images as a gradual weakening and eventual disappearance of the ablation phenomena.

### 3.3. Influence of Millisecond Laser Peak Power on Oblique Hole Processing Performance

**Figure 6:**
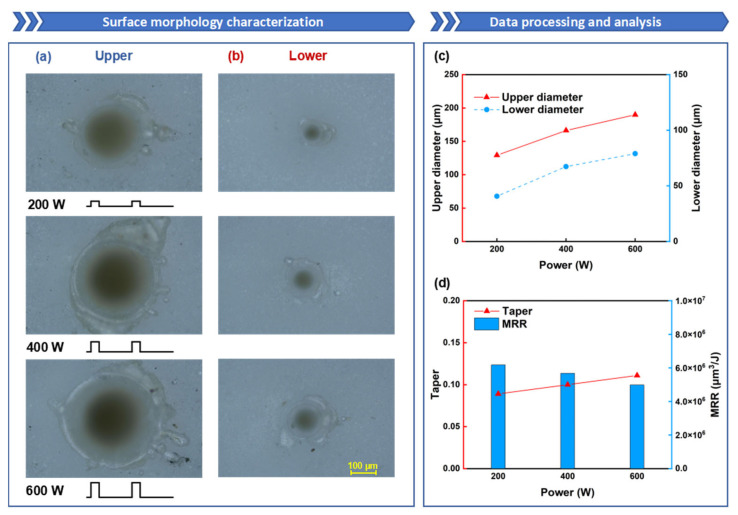
The influence of power on oblique hole processing performance: the laser power is set to 200 W, 400 W, and 600 W, while the other parameters are fixed: ablation time of 100 ms, duty cycle of 20%, and repetition rate of 100 Hz: (**a**) Upper-surface morphology; (**b**) Lower-surface morphology; (**c**) Upper- and lower-surface diameter line chart; (**d**) Taper and ablation efficiency comparison.

Secondly, the influence of millisecond laser peak power on the quality of hole processing and the forming process is investigated. Figure 6a,b depict the morphology of the upper and lower surfaces at different peak power levels. An increase in power signifies a rise in laser energy density both temporally and spatially, which leads to a faster ablation rate and larger ablation areas.

As illustrated in Figure 6c, it is evident that as the laser power increases, the diameters of both the upper and lower surfaces expand accordingly; the diameter of the upper surface increased from 129 μm to 190 μm, and the diameter of the lower surface increased from 41 μm to 79 μm. This phenomenon is attributed to the distribution characteristics of the Gaussian beam; when the peak power of the millisecond laser escalates, the overall laser flux of the focused beam increases, enabling the laser to heat and melt the alumina ceramic over a larger diameter range. From Figure 6d, it can be observed that as the power rises, the taper of the hole deteriorates and the MRR decreases. According to the formula for taper, since the thickness of the sample remains constant, the difference in diameters between the upper and lower surfaces determines the taper of the hole. As the power increases, although both the upper and lower diameters increase, the difference between them becomes progressively larger, hence increasing the power results in a poorer hole taper. During the hole formation stage, as the depth of the hole increases, the laser energy reflected multiple times inside the hole becomes increasingly difficult to escape from the ablation zone, and most of the energy is confined within the ablation zone until it is absorbed. When a through hole is formed, the laser energy escaping from the hole increases, thus the MRR is highest precisely when the through hole is formed. At a power of 200 W, the alumina ceramic sample is already penetrated, at which point the MRR is at its peak.

**Figure 7:**
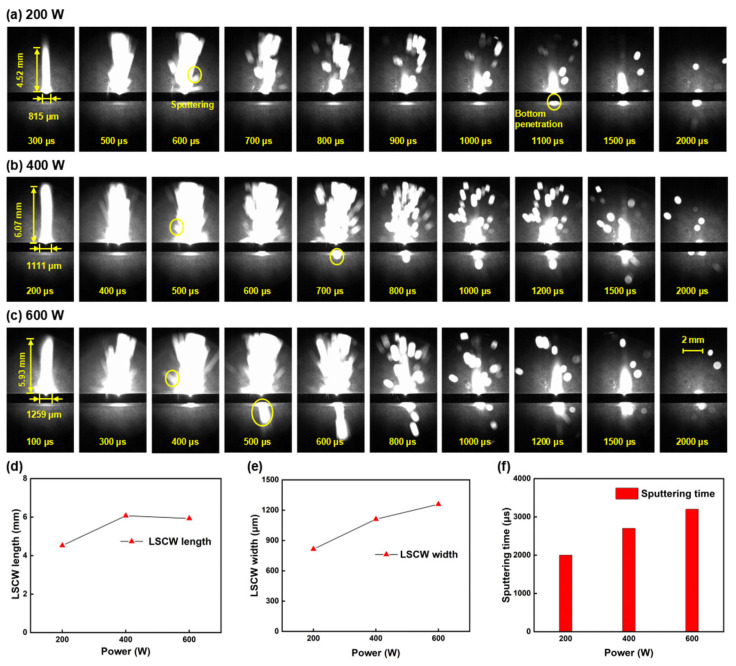
Shadow sequence images of alumina ceramic processed by millisecond lasers at different power levels: (**a**) 200 W; (**b**) 400 W; (**c**) 600 W; (**d**) LSCW length line chart; (**e**) LSCW width line chart; (**f**) Sputtering time bar chart.

High-speed shadow imaging technology is employed to meticulously analyze the ablation process at various power levels, with Figure 7a–c showcasing the shadow sequence images for each power level. The shadow sequence images are utilized to investigate the influence of peak power on the preheating duration, the morphology of the LSCW, and the sputtering phenomena. From the shadow sequence images, it is observed that increasing the power slightly shortens the preheating stage of the sample. The preheating duration decreases from 500 μs at 200 W to 400 μs at 400 W and further to 300 μs at 600 W. Figure 7d–f present line charts derived from the ablation process data shown in Figure 7a–c, revealing that as the power increases, the width of the LSCW gradually expands, while its height appears to saturate around 6 mm. This saturation in height may arise from the enhanced flux of the Gaussian laser beam due to higher average power, which enlarges the ablation area on the sample surface and increases instantaneous energy deposition, thereby broadening the LSCW width. However, the LSCW height is constrained by the magnitude of recoil pressure, leading to height saturation. The sputtering phenomena during ablation also intensify with higher power. Increased power elevates the energy input into the sample, raising its processing temperature. Consequently, higher temperatures generate larger and more sustained vapor pressures, prolonging and intensifying material sputtering. Additionally, the shadow sequence images show no observable sputtering at the bottom for 200 W, indicating incomplete through-hole formation or a smaller lower-surface diameter at this power level, consistent with prior hole morphology data. At 400 W, bottom sputtering becomes visible at 800 μs, while at 600 W, sputtering occurs as early as 500 μs. This demonstrates that higher laser power enhances penetration and ablation capabilities.

### 3.4. Influence of Millisecond Laser Ablation Time on Oblique Hole Processing Performance

**Figure 8:**
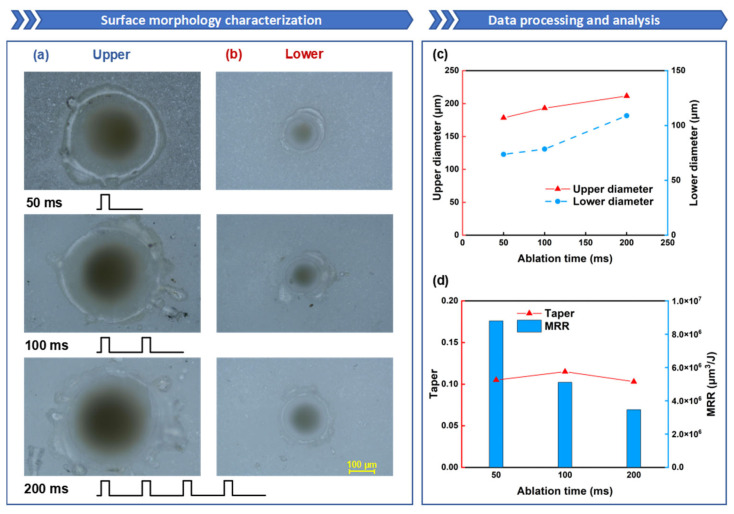
The influence of ablation time on oblique hole processing performance: the ablation time is set to 50 ms, 100 ms, and 200 ms, while the other parameters are fixed: power of 600 W, duty cycle of 20%, and repetition rate of 100 Hz: (**a**) Upper-surface morphology; (**b**) Lower-surface morphology; (**c**) Upper- and lower-surface diameter line chart; (**d**) Taper and ablation efficiency comparison.

Subsequently, the impact of ablation time on the ablation morphology is investigated, and the corresponding dynamic ablation process is analyzed. Figure 8a,b depict the morphology of the upper and lower surfaces at different ablation times. The essence of increasing ablation time is to augment the number of millisecond laser pulses. Previous pulse lasers cause damage to the alumina ceramic, and on this basis, the absorptivity of the alumina ceramic to subsequent pulse energy increases, which further raises the temperature of the alumina ceramic, even reaching the evaporation temperature. Under the influence of saturated vapor pressure and heat transfer, the size of the hole will increase with an increase in ablation time.

As shown in Figure 8c, as the ablation time increases, the diameters of both the upper and lower surfaces exhibit a gradual increasing trend. Notably, the variation in the lower-surface diameter with ablation time is more pronounced. The diameter of the upper surface increased from 178 μm to 212 μm, and the diameter of the lower surface increased from 74 μm to 109 μm. After through-hole formation, a greater proportion of laser energy escapes from the hole through wall reflections rather than contributing to further ablation, leaving material removal entirely dependent on heat transfer effects. With prolonged ablation time, subsequent pulse energy continuously accumulates within the alumina ceramic. The molten material at the bottom is progressively removed under the combined action of saturated vapor pressure and gravity, leading to gradual expansion of the lower diameter. Figure 8d demonstrates no clear correlation between ablation time and hole taper. Through-hole formation is achieved at 50 ms of laser ablation time, reaching peak MRR of 8.80 × 10^6^ μm^3^/J. However, as the ablation time extends, the MRR progressively declines.

**Figure 9:**
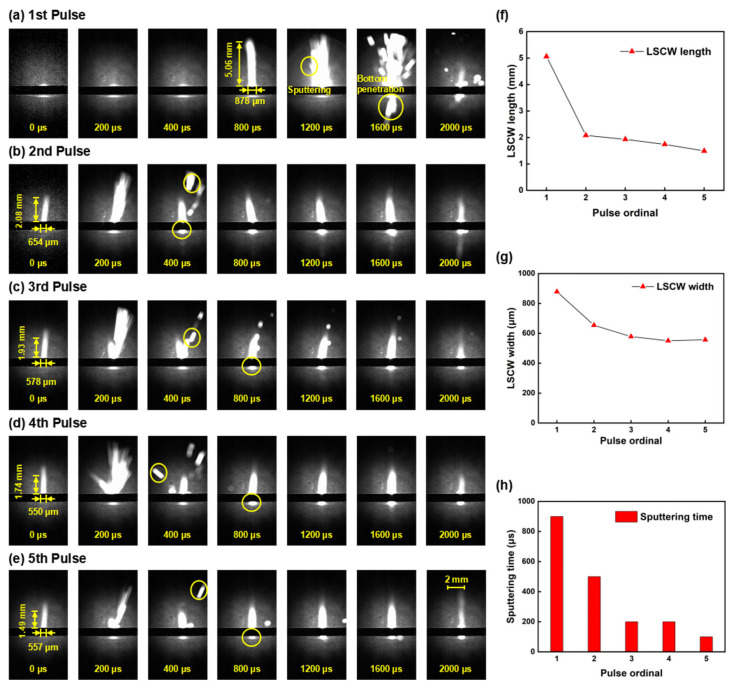
Shadow sequence images of alumina ceramic processed by millisecond lasers at different ablation time: (**a**) 1st pulse; (**b**) 2nd pulse; (**c**) 3rd pulse; (**d**) 4th pulse; (**e**) 5th pulse; (**f**) LSCW length line chart; (**g**) LSCW width line chart; (**h**) Sputtering time bar chart.

To clearly demonstrate the differences in preheating times across various pulses, the coating on the sample is made thinner compared to other samples, slightly reducing the surface absorptivity to extend the preheating duration. Observing the shadow sequence images, the operation and shutdown processes of the millisecond laser are distinctly visible. Each millisecond pulse lasts 2 ms, with a working cycle of 10 ms, totaling 50 ms of operation and 5 pulses. In the first pulse image, Figure 9a, within 800 μs, only the laser acting on the alumina ceramic sheet is observable, displaying a semicircular bright area in the image. This indicates the initial stage of ablation, where the material surface, aided by the black coating, absorbs laser energy and gradually melts to form a molten pool. At a delay of 900 μs, a slender combustion LSCW is clearly visible on the alumina ceramic surface under laser action, approximately 5.06 mm in height and 878 μm in width, but no material sputtering is observed at this stage. This phase is the preheating stage. As the delay extends, sputtering phenomena gradually appear on the material surface. By 1100 μs, a significant amount of ejecta is visible, marking the transition into the intense sputtering phase of the millisecond laser interaction with alumina ceramic. The intense sputtering phase lasts until 1600 μs, after which the ejecta noticeably decrease, and only sporadic ejecta are observed until the end of the first pulse, indicating the material has entered the stable ablation phase. Sputtering phenomena at the bottom of the alumina ceramic sheet are observable after a delay of 1400 μs, suggesting that a through hole has formed on the alumina ceramic sheet under the first pulse. In the second pulse image, Figure 9b, due to the damage caused by the first pulse to the alumina ceramic surface, the absorptivity of the alumina ceramic to subsequent pulse energy significantly increases, greatly reducing the preheating time. The generation of the LSCW is observable at a pulse delay (the delay since the last pulse) of 0 μs, but its height is reduced compared to the first pulse. Sputtering phenomena become visible after a pulse delay of 200 μs, lasting approximately 200 μs, after which the ablation enters the stable ablation phase, where only the combustion LSCW is visible without sputtering phenomena. The phenomena in the subsequent three pulses are essentially consistent, as shown in Figure 9c–e, with the LSCW generated at 0 μs and sputtering phenomena starting after 200 μs, with reduced duration and intensity of sputtering. Although there is a slight decreasing trend in the morphology of the LSCW and the intensity of sputtering from the second to the fourth pulse, the changes are minimal. By the fifth pulse, the LSCW significantly shrinks, and the sputtering phenomena become faint, indicating that most of the material in the hole has been removed.

Figure 9f–h present line charts derived from the ablation process data illustrated in Figure 9a–e, showing that as the number of pulses increases, the height and width of the LSCW, as well as the duration of sputtering, gradually decrease. The decrease from the first to the second pulse is particularly pronounced, with subsequent data showing a gradual but less significant decline.

Analysis of shadowgraph imaging data reveals the mechanism behind decreasing MRR with prolonged ablation time. After through-hole formation in the alumina ceramic, most laser energy penetrates the sample directly. The material removal mechanism consequently shifts from laser ablation to heat conduction-dominated processes, significantly reducing removal efficiency. High-speed imaging confirms perforation completion within hundreds of microseconds. Therefore, when calculating MRR, the total energy input decreases progressively over time [57].

### 3.5. Influence of Millisecond Laser Duty Cycle on Oblique Hole Processing Performance

**Figure 10:**
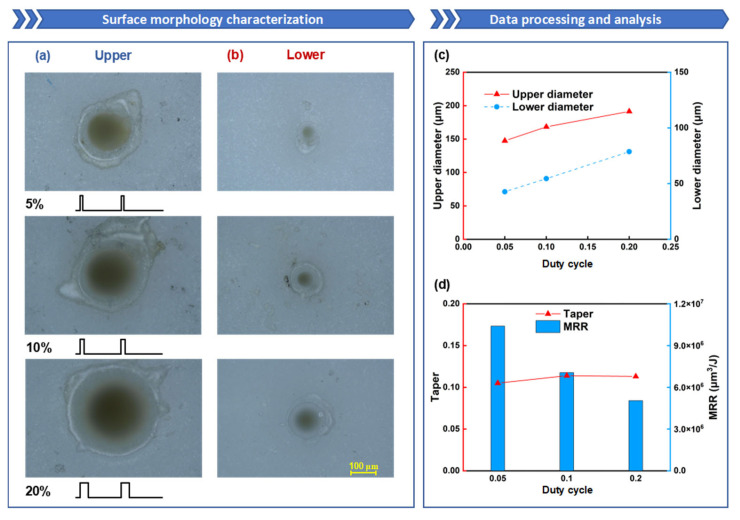
The influence of duty cycle on oblique hole processing performance: the duty cycle is set to 5%, 10%, and 20%, while the other parameters are fixed: power of 600 W, ablation time of 100 ms, and repetition rate of 100 Hz: (**a**) Upper-surface morphology; (**b**) Lower-surface morphology; (**c**) Upper- and lower-surface diameter line chart; (**d**) Taper and ablation efficiency comparison.

Finally, the influence of the duty cycle is investigated. Figure 10a,b depict the morphology of the upper and lower surfaces at different duty cycles. An increase in the duty cycle implies greater single-pulse energy and shorter cooling times, which facilitates the accumulation of laser energy. Compared to low duty cycles, high duty cycles generate larger saturated vapor pressures and heat transfer effects, resulting in the removal of more molten alumina. Consequently, the diameters of both the upper and lower surfaces increase with the duty cycle; the diameter of the upper surface increased from 147 μm to 191 μm, and the diameter of the lower surface increased from 43 μm to 79 μm, as shown in Figure 10c.

From Figure 10d, it can be observed that as the duty cycle increases, the taper gradually rises, but the rate of increase is relatively small. The taper under all three conditions remains around 1:10. When the duty cycle is increased, the single-pulse energy rises, and the material cooling time decreases. This means that increasing the duty cycle exacerbates thermal effects, leading to a deterioration in hole taper. However, increasing the single-pulse energy also enhances the penetration and ablation capabilities of the millisecond laser. This implies that as the duty cycle increases, the lower-surface hole diameter expands rapidly, which partially offsets the adverse thermal effects of a high duty cycle. As a result, the taper increases slowly with the duty cycle, especially when the duty cycle rises from 10% to 20%, where the taper shows a decreasing trend. Comparing the ablation efficiencies under different duty cycles, the highest MRR of 1.04 × 10^7^ μm^3^/J is achieved at a duty cycle of 5%. From Figure 10a, it can be seen that when the duty cycle is 5%, a through hole has already been formed. In the case where a through hole has already been formed, the lower the total energy, the less energy escapes. Therefore, the MRR is highest at a duty cycle of 5%.

**Figure 11:**
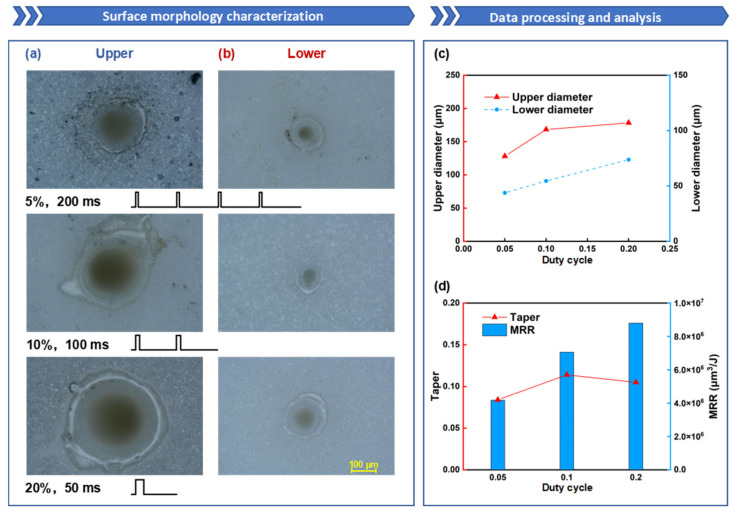
The influence of duty cycle on oblique hole processing performance: the duty cycle is set to 5%, 10%, and 20%, with corresponding ablation times of 200 ms, 100 ms, and 50 ms, while the other parameters are fixed: power of 600 W and repetition rate of 100 Hz: (**a**) Upper-surface morphology; (**b**) Lower-surface morphology; (**c**) Upper- and lower-surface diameter line chart; (**d**) Taper and ablation efficiency comparison.

To further compare the drilling data under the same energy but different duty cycles, as shown in Figure 11a–d, the variation in hole taper is observed. It is found that under the same total laser energy, the change in taper is more pronounced, with the taper first increasing and then decreasing as the duty cycle rises. Comparing this with the previous data, it is noted that when the duty cycle is 5%, the upper-surface diameter of the hole decreases as the ablation time increases. This may be due to the lower single-pulse energy at a 5% duty cycle, resulting in insufficient recoil pressure. As the ablation time increases, more molten material accumulates inside the hole. This molten material flows to the hole entrance under the recoil pressure and forms a recast layer upon cooling, leading to hole closure. From Figure 11c, it can be seen that the hole diameter steadily increases with the duty cycle, particularly evident in the change in the lower-surface diameter. This indicates that increasing the duty cycle under the same total energy can effectively enhance material removal efficiency, especially the ablation capability at the material’s bottom. This suggests that when the total energy remains constant, appropriately increasing the duty cycle can improve hole taper and MRR, as shown in Figure 11d. However, excessively high single-pulse energy can cause severe thermal effects, leading to issues such as an overly large ablation area and excessive cracks within the ablation zone, significantly affecting hole processing quality. Therefore, during millisecond laser drilling, it is advisable to moderately increase the duty cycle to improve hole taper and MRR.

**Figure 12:**
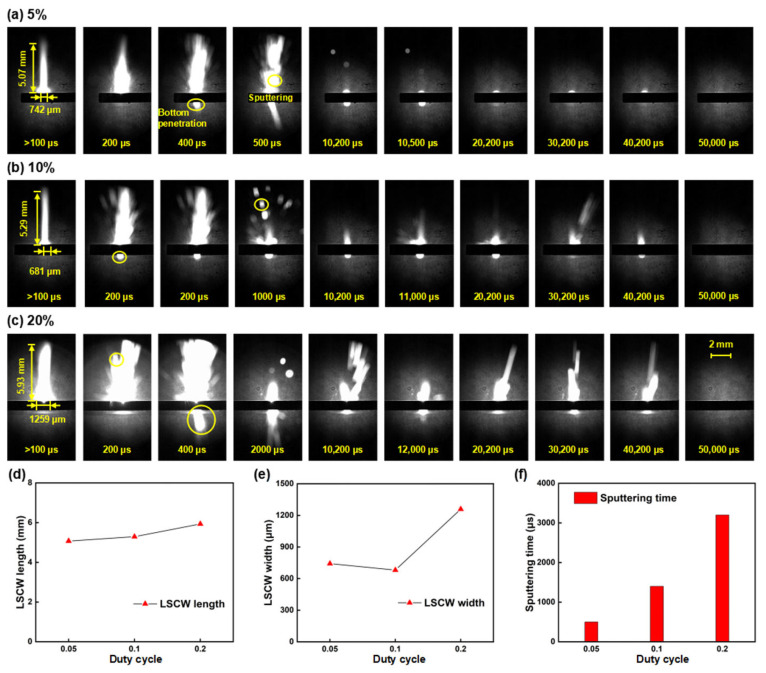
Shadow sequence images of alumina ceramic processed by millisecond lasers at different duty cycles: (**a**) 5%; (**b**) 10%; (**c**) 20%; (**d**) LSCW length line chart; (**e**) LSCW width line chart; (**f**) Sputtering time bar chart.

Figure 12a–c present shadow sequence images at different duty cycles. By comparing the differences in ablation phenomena across various duty cycles, it is found that the morphology of the LSCW at 10% and 5% duty cycles is quite similar, indicating that under the same laser power, changes in the single-pulse duration have little effect on the morphology of the LSCW. From Figure 12d,e, it can be seen that the LSCW at a 20% duty cycle is significantly more robust, possibly due to uneven application of the black coating, resulting in higher absorption in certain areas and thus greater absorbed laser energy, which produces a more robust LSCW. Furthermore, from the microscopic morphology images at different duty cycles, it is evident that the higher the duty cycle, the larger the ablation area. This is because as the duty cycle increases, the alumina ceramic more easily accumulates laser energy, and under the influence of heat transfer, the molten pool structure at higher duty cycles further expands. Comparing the sputtering phenomena across different duty cycles, since the power is the same, sputtering phenomena occur at 200 μs in all three conditions, with bottom sputtering starting at 400 μs and continuing until the end of the first pulse. In subsequent pulses, the 20% duty cycle, with its higher single-pulse energy, almost always exhibits sputtering phenomena; the 10% duty cycle occasionally shows sputtering phenomena, and the 5% duty cycle rarely shows any sputtering phenomena. This may be because subsequent pulses lack the coating-assisted ablation, requiring more laser energy to produce sputtering phenomena, and the existing hole depth further complicates the observation of sputtering phenomena. Under these conditions, the 5% duty cycle, with its lower single-pulse energy, rarely exhibits sputtering phenomena in subsequent pulses. From Figure 12f, it can be concluded that both the duration and intensity of sputtering are positively correlated with the duty cycle, supporting the view that increasing single-pulse energy effectively enhances material removal efficiency under the same total energy.

## 4. Discussion

**Figure 13:**
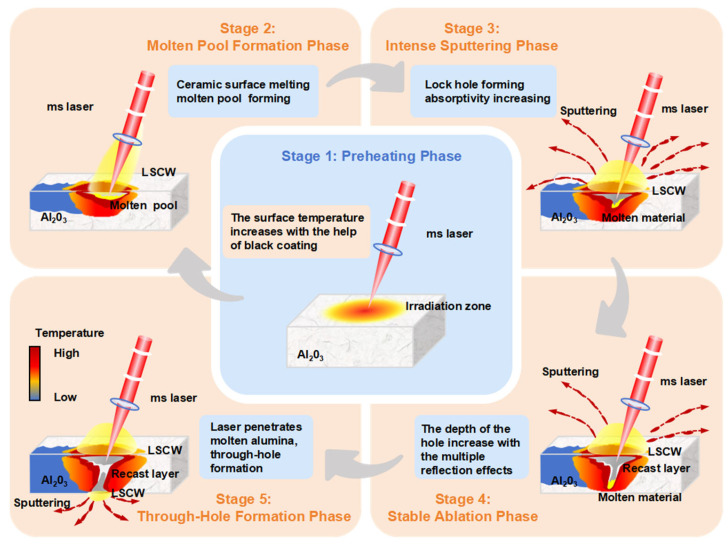
Schematic diagram of millisecond laser oblique hole formation. The diagram illustrates the five stages of millisecond laser oblique hole drilling in alumina ceramics. The central blue zone represents a schematic of Preheating Phase. The surrounding orange zones depict the material removal process, corresponding to Molten Pool Formation Phase, Intense Ablation Phase, Stable Ablation Phase, and Through-Hole Formation Phase.

Following the completion of process experiments, we obtained insights into the effects of various laser parameters on oblique hole morphology and material removal dynamics. In this section, we discuss the material removal mechanisms in millisecond laser oblique hole processing of alumina ceramics by drawing on results from simulations, imaging, and experimental analyses. Figure 13 systematically illustrates millisecond laser oblique hole processing in alumina ceramics, dividing the evolution of hole formation into five stages.

As shown in Figure 13, the millisecond laser hole formation process in alumina ceramic can be divided into five stages. Stage 1: Preheating Phase. The millisecond laser begins ablating the sample with the assistance of the surface coating, and the surface temperature gradually rises. Stage 2: Molten Pool Formation Phase. When the surface temperature reaches the melting point of alumina ceramic, a molten pool forms, and an LSCW becomes observable on the sample surface. Stage 3: Intense Sputtering Phase. After molten pool formation, the laser absorptivity of the alumina ceramic increases rapidly, causing a sharp rise in energy within the molten pool over a short period. Under the recoil vapor pressure, intense sputtering occurs, rapidly deepening the molten pool and forming the initial hole morphology. Stage 4: Stable Ablation Phase. The hole reaches a certain depth, and the laser undergoes multiple reflections within the hole, significantly enhancing material absorption. Molten material continues to be removed by recoil vapor pressure, but as the hole deepens, not all molten material can be expelled. Some accumulates near the hole entrance, forming a recast layer, and the sputtering intensity diminishes. Due to the high transparency of molten alumina to the laser, the millisecond laser penetrates the recast layer and continues deepening the hole. Stage 5: Through-Hole Formation Phase. The sample is fully penetrated. At this stage, the recoil pressure direction aligns with gravity, and molten material accumulated at the hole bottom is ejected from the sample underside. After through-hole formation, most laser energy escapes from the hole, and further diameter expansion relies solely on heat transfer, with no observable sputtering phenomena. Notably, due to enhanced absorption, subsequent laser pulses (starting from the second pulse) generate LSCWs immediately upon irradiation. If a through hole already exists, subsequent laser–material interactions primarily involve Stages 2 and 5, with minimal or no sputtering phenomena.

## 5. Conclusions

This paper integrates numerical simulation models, high temporal resolution shadow imaging, and process experiments to systematically explore the dynamic processes and mechanisms of oblique hole processing with millisecond lasers, providing guidance for researching optimization methods of hole quality. The primary conclusions are as follows:

(1) A combined approach integrating simulation, imaging, and experimental analysis is employed to investigate oblique hole processing and its quality control. This study systematically elucidates the phenomena of temperature variations, molten pool dynamics, and material removal mechanisms during the interaction between millisecond lasers and alumina ceramics. These interrelated phenomena collectively summarize the dynamic process of hole formation in alumina ceramics via millisecond laser processing.
(2) The numerical simulation model for oblique hole processing in alumina ceramic using millisecond lasers has been established. By utilizing this model to investigate the variations in temperature, molten pool morphology, and hole depth during millisecond laser oblique hole processing under different repetition rates, it provides critical guidance for the selection of laser parameters in subsequent process experiments.
(3) For the first time, the high-temporal-resolution shadowgraph imaging technique has been employed to characterize the material removal phenomena on the upper and lower surfaces of alumina ceramic during millisecond laser oblique hole processing. The influence of different laser parameters on the ablation process is investigated. It is concluded that the morphology of the LSCW is most significantly related to power and becomes finer with increasing pulse number, and that the material removal process is mainly concentrated in the first pulse phase.
(4) Systematically studies the influence of different laser parameters on the morphology of oblique holes. Experiments using a 40 Hz ms laser achieve an ablation efficiency of 9.44 × 10^6^ μm^3^/J and a hole with a taper of 1:11. It is concluded that increasing the energy per pulse can effectively optimize ablation efficiency and achieve better penetration ablation performance.

In summary, this work provides practical guidance for research on long-pulse laser drilling. The optimized shadowgraph imaging technique enables a more comprehensive understanding of material removal mechanisms, while the integrated theoretical–imaging–experimental methodology offers a novel approach for subsequent studies on laser drilling processes.

## Figures and Tables

**Table 1:** Alumina ceramic properties for the simulation [66].

Property	Symbol	Value (Alumina)
Solid density	$\rho_{s}$	3720 (kg/m^3^)
Liquid density	$\rho_{l}$	3200 (kg/m^3^)
Solid thermal conductivity	$k_{s}$	25 (W/(m∙K))
Liquid thermal conductivity	$k_{l}$	15 (W/(m∙K))
Solid-specific heat	$C_{s}$	880 (J/(kg∙K))
Liquid-specific heat	$C_{l}$	1257 (J/(kg∙K))
Melting point	$T_{m}$	2350 (K)
Vaporization point	$T_{v}$	3280 (K)
Latent heat of melting	$L_{m}$	1.06743 × 10^6^ (J/kg)
Latent heat of vaporization	$L_{v}$	1.0665 × 10^6^ (J/kg)
Convective heat transfer coefficient	h	20 (W/(m^2^∙K))
Dynamic viscosity	u	0.05 (Pa∙s)
Surface tension coefficient	σ	0.02 (N/m)
Absorptivity	$\alpha_{ms}$	0.27
Radiation emissivity	$\sigma_{0}$	0.2
Boltzmann constant	$k_{B}$	1.380649 × 10^−23^ (J·K^−1^)

**Table 2:** The laser parameters for the experiment.

Parameter	Range
Peak power, *P* (W)	200 ≤ *P* ≤ 600
Puls repetition rate, *f* (Hz)	40 ≤ *f* ≤ 500
Puls duty cycle, *D* (%)	5 ≤ *D* ≤ 20
Puls duration, *t_on_* (ms)	50 ≤ *t_on_* ≤ 200

## Data Availability

The original contributions presented in the study are included in the article; further inquiries can be directed to the corresponding authors.
